# Management of patients with magnetically controlled growth rods amidst the global COVID-19 pandemic

**DOI:** 10.1007/s00586-020-06516-2

**Published:** 2020-06-29

**Authors:** Harry Hothi, Stewart Tucker, Masood Shafafy, Colin Nnadi, Kenneth M. C. Cheung, Elisabetta Dal Gal, Martina Tognini, Johann Henckel, John Skinner, Alister Hart

**Affiliations:** 1 The Royal National Orthopaedic Hospital and Institute of Orthopaedics and Musculoskeletal Science, University College London, Brockley Hill, Stanmore, HA74LP UK; 2 Great Ormond Street Hospital for Children, NHS Foundation Trust, London, UK; 3 Department of Trauma and Orthopaedics, Nottingham University Hospitals NHS Trust, Nottingham, UK; 4 Nuffield Orthopaedic Centre, Oxford University Hospital, Headington, Oxford UK; 5 Department of Orthopaedics and Traumatology, The University of Hong Kong, 102 Pokfulam Road, Pokfulam, Hong Kong SAR China

**Keywords:** COVID-19, MAGEC rod, MCGR, EOS

## Abstract

**Introduction:**

At the time of writing, we are all coping with the global COVID-19 pandemic. Amongst other things, this has had a significant impact on postponing virtually all routine clinic visits and elective surgeries. Concurrently, the Magnetic Expansion Control (MAGEC) rod has been issued with a number of field safety notices and UK regulator medical device alerts.

**Methods:**

This document serves to provide an overview of the current situation regarding the use of MAGEC rods, primarily in the UK, and the impact that the pandemic has had on the management of patients with these rods.

**Results and Conclusion:**

The care of each patient must of course be determined on an individual basis; however, the experience of the authors is that a short delay in scheduled distractions and clinic visits will not adversely impact patient treatment. The authors caution against a gap in distractions of longer than 6 months and emphasise the importance of continued remote patient monitoring to identify those who may need to be seen more urgently.

## Background to MCGRs

MCGRs are used in the surgical treatment of children with scoliosis; the rods serve to brace the spine and minimise the progression of scoliosis as the child grows. An external magnet is used to extend the length (distract) of the rods, in-line with the growth of the child; this is performed at regular intervals, usually between 1 and 6 months in a routine outpatient ‘distraction clinic’ visit. These rods are intended to be removed after they have been extended to their full length; these may be replaced with longer rods if the patient is still growing or the patient may undergo other treatment options if growth has stopped.

In the UK, one design of MCGR has been available for clinical use, known as the MAGnetic Expansion Control (MAGEC) rod (NuVasive). Since its first use in 2009, there have been 7 design iterations of the MAGEC rod, namely: MAGEC 1.0, 1.1, 1.2, 1.3, 2.0, 2.1 and most recently the MAGEC X (first used mid-2017).

## What are the known issues?

On the 1 of April 2020, the manufacturer issued an FSN, voluntarily suspending the supply of all MAGEC rods to the UK, and the MHRA released an MDA the same day confirming this with the action that surgeons in the UK should not implant MAGEC rods until further notice [1].

The MHRA is now investigating whether the clinical benefits of using these rods continue to outweigh the risks. The regulator will in exceptional circumstances still consider use of MAGEC in patients during this period on a case-by-case basis.

This action comes as a result of previous FSNs and MDAs [2, 3] highlighting issues with a fracture/failure of external and internal components and the generation of titanium wear/corrosion debris [4], in some cases preventing rod distractions, ultimately requiring early and unplanned revision, as in Table 1.

**Table 1:** Summary of the issues and clinical risks associated with MAGEC rod designs

Design iteration	Implant issue	Clinical risk
MAGEC X	Risk of a separation of the threaded end cap from the housing tube after implantation	Internal components may be exposed to biological fluid, potentially leading to a failure of the mechanism and the release of titanium wear and corrosion debris
MAGEC 1.0, 1.1, 1.2 (i.e. rods manufactured before 26 March 2015)	These rods have an increased risk of a fracture of the internal locking pin. There is also evidence of a failure of the O-ring seal in some rods and the generation of titanium wear/corrosion debris	A fractured locking pin may prevent the rod from lengthening. There is no evidence on long-term effect of the sometimes-significant debris in these children; however, excessive debris may also prevent the rod from extending and lead to discolouration of surrounding tissue

The availability and use of these devices in other countries has currently seen no change as a result of action in the UK; MAGEC 1.3, 2.0 and 2.1 continue to be implanted routinely.

**Fig. 1:**
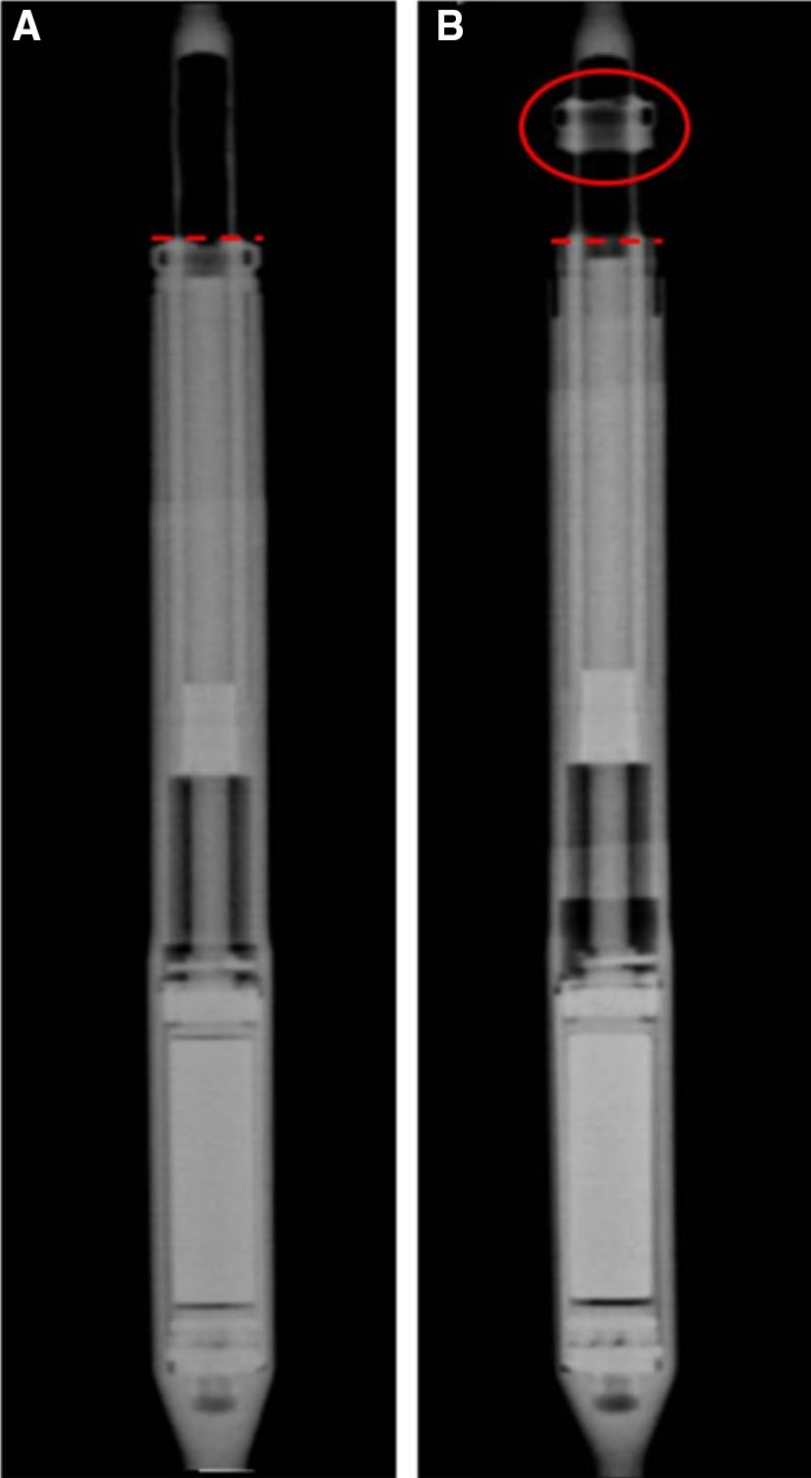
Example X-ray images of (**a**) a rod with a well-fixed end cap and (**b**) a rod with a separated end cap [3]

The exception is the MAGEC X edition, which was recalled globally by the manufacturer due to the risk of end-cap separation (Fig. 1); the manufacturer has stated, however, that the rods may still continue to distract or serve as an internal brace even if end-cap separation has occurred. Furthermore, given that current information states that this issue has occurred in 0.5% of patients with this design, the risk of immediate adverse impacts appears low.

The most common mode of device failure reported in the literature for previous rod designs (i.e. those other than MAGEC X) had been a fracture of the internal locking pin, resulting in an inability for the rod to be lengthened further; the manufacturer reports a fracture risk of 5%. Similarly, the rods may still function as an internal brace until they can be revised at an appropriate time.

Surgeons will of course have two primary considerations amidst these alerts and the pandemic:

1. Identifying which (if any) of their patients have experienced (or are at risk of experiencing) the implant issues described above, so as to appropriately manage them.
2. Continuing with timely rod distractions, in-line with the growth of the child.

## What do the normal guidelines say?

The MHRA advises that surgeons should notify all patients about the possible complications that may occur due to device failures. Surgeons should continue to use their own clinical judgement to assess each patient individually and perform X-ray imaging (and not ultrasound) at least once every 6 months. Patients with the MAGEC X should have X-ray imaging performed within 3 months of the alert date of 18 March 2020 to do determine if cap separation has occurred, as in Fig. 1.

Under the normal pathway, patients would attend an outpatient distraction clinic every 1–6 months during which the surgeon/clinical team could discuss these issues, carry out clinical assessments in person and perform a routine rod lengthening. These visits could also coincide with X-ray imaging as suggested by the MHRA.

## What do we do in a COVID-19 environment?

The situation with COVID-19 is fast evolving; at the time of writing, the UK remains under a lockdown to slow down the spread of the virus with all routine clinic visits and elective surgeries postponed. In contrast, Hong Kong is still practicing containment measures meaning that some reduced clinics are still able to run but every patient is risk assessed for having COVID-19 before they are seen.

In light of these circumstances, the suspension of the supply of MAGEC rods in the UK, and the necessity for all MAGEC patients to undergo timely distractions, we offer the following considerations to surgeons, parents and patients in the UK (Table 2).

**Table 2:** Summary of current guidance and suggested changes under COVID-19

Design iteration	Normal guidance	Under COVID-19
MAGEC X	Anteroposterior X-ray imaging within 3 months of the original alert on 18 March 2020 to determine if cap separation has occurred (Fig. 1)	X-ray and clinical assessment when it is safe and practical to do so. Acknowledgement that this is likely to be beyond the timeframe suggested by the MHRA. Titanium blood tests may be a useful surrogate for implant performance (relating to wear/corrosion) in the interim, however, needs further research
All MAGEC rods	Advise all patients about the possible complications resulting from the failure of components as described in previous FSNs. Each patient should be assessed using own clinical judgement and using X-ray imaging (rather than ultrasound) at least once every 6 months	
All MAGEC rods	Distraction clinic every 3–6 months	Distraction interval of every 6 months if possible. Earlier if child experiences mild discomfort

###  Determining any adverse impact of end-cap separation or other device failures (including all MAGEC rod designs implanted)

Routine clinic visits and X-ray imaging should resume when it is safe and practical to do so within the confines of social distancing measures. As acknowledged by the MHRA in their MDA on 1 April 2020 [1], it is likely that the follow-up of these patients will not be possible within the timeframe that they have suggested due to the pandemic.

The occurrence of an end-cap separation in the MAGEC X can only be determined following X-ray imaging at a timepoint that is practically safe and possible.

Surgeon experience in these cases is that the inevitable delay in the normal follow-up pathway will not adversely impact the majority of patients. Regular and remote (i.e. telephone) monitoring of patient comfort may be the best way to identify patients that may need to be seen urgently.

As an additional consideration, there is evidence of titanium debris being released from the devices due to the failure modes described above [4]. It is proposed that measures of titanium levels in blood samples from MAGEC patients may be a useful additional monitoring tool if it is practically possible and the appropriate collection, storage and analysis protocols can be utilised without impacting any resources required for the management of the pandemic [5]. This, however, requires further research to better understand the sensitivity and specificity of this measure.

###  The appropriate time between distraction clinics in light of COVID-19

Under normal circumstances, a distraction frequency of once every 1–6 months is typical. Under the current circumstances, it is likely that all distractions (where possible) will have to be delayed to a 6-month frequency with the expectation that we will have passed the peak of this pandemic by then.

In the clinical experience of the treating MAGEC surgeons, the majority of patients will not be impacted by a short delay in their distractions; however, there should not be a gap of more than 6 months unless in exceptional circumstances. The surgeon and/or clinical nurse specialists will remain in touch with patients remotely. Some children will, however, experience mild discomfort indicative that the rod requires lengthening or there may be obvious curve progression; these patients may be seen sooner, and lengthening brought forward. Once the situation returns to normal, surgeons can shorten significantly the gap between lengthenings if needed to catch up with a patient’s growth.

In circumstances in which surgery is inevitably required to revise a failed implant or indeed if a planned removal is necessary to facilitate final fusion, a senior clinician within the framework of a Multidisciplinary Team (MDT) needs to have a balanced discussion about the risk of COVID transmission with the patient and family regarding suitability of proceeding imminently or delaying definitive surgery to a later date.

As countries begin to lift lockdown measures, MDT decision making will be effective at managing the return to normality in terms of patient management, whilst being mindful that some risk from the virus may remain for a considerable time yet.

## Conclusion

There is currently much uncertainty regarding the use of MCGRs in the UK due to recent regulator MDAs. The management of these patients has been further complicated by the current COVID-19 pandemic. The care of each patient must of course be determined on an individual basis; however, the experience of the authors is that a short delay in scheduled distractions and clinic visits will not adversely impact patient treatment. The authors caution against a gap in distractions of longer than 6 months and emphasise the importance of continued remote patient monitoring to identify those who may need to be seen more urgently.
